# Supplementary material for: Work autonomy and its associated factors among nurses working in South Gondar zone public hospitals, Amhara regional state, Northcentral Ethiopia: Institution-based cross-sectional study
Source: PLoS One. 2026 Feb 13;21(2):e0339208. doi: 10.1371/journal.pone.0339208 (PMC12904413; doi:10.1371/journal.pone.0339208)
Supplement: Attachment — Submitted filename: POINT - new.pdf [file pone.0339208.s002.pdf]

Response to Reviewers

We appreciate the time and effort that the editors and the reviewers dedicated to providing feedback on our manuscript and are grateful for the insightful comments and valuable improvements to our paper. We have incorporated all suggestions made by the editors and the reviewers. Please see below for a point-by-point response to the reviewers' comments and concerns. We are thankful to the reviewer for this valuable suggestion.

Point-by-point reply to the editors' comments

1. Please ensure that your manuscript meets PLOS ONE's style requirements, including those for file naming. The PLOS ONE style templates can be found at

https://journals.plos.org/plosone/s/file?id=wjVg/PLOSOne_formatting_sample_main_body.pdf
and
https://journals.plos.org/plosone/s/file?id=ba62/PLOSOne_formatting_sample_title_authors_affiliations.pdf

RESPONSE: Thank you for your insightful recommendations. We prepared the title/authors/affiliations page and main body of the manuscript in accordance with the PLOS ONE style templates. Tables/figures are arranged and quoted sequentially.

2. Please provide additional details regarding participant consent. In the ethics statement in the Methods and online submission information, please ensure that you have specified (1) whether consent was informed and (2) what type you obtained (for instance, written or verbal, and if verbal, how it was documented and witnessed). If your study included minors, state whether you obtained consent from parents or guardians. If the need for consent was waived by the ethics committee, please include this information.

RESPONSE: Thank you for your insightful recommendations. We expanded the ethics statement in the methods section and updated the online submission information to clearly indicate that written informed consent was obtained from the study participants before the study commencement. The study did not include any names or personally identifiable information.

3. Your ethics statement should only appear in the Methods section of your manuscript. If your ethics statement is written in any section besides the Methods, please delete it from any other section.

RESPONSE: Thank you for your insightful recommendations. The ethics statement now appears only in the Methods section and has been removed from all other sections of the manuscript.

4. Please ensure that you refer to Figure 1 in your text as, if accepted, production will need this reference to link the reader to the figure.

RESPONSE: Thank you for your insightful recommendations. We inserted Figure 1 in the text, ensuring it appears in the correct numerical order in the manuscript.

5. We note you have included a table to which you do not refer in the text of your manuscript. Please ensure that you refer to Tables 1 and 3 in your text; if accepted, production will need this reference to link the reader to the tables.

RESPONSE: Thank you for your insightful recommendations. We inserted explicit in-text citations for Tables 1 and 3 at their first mention in the results section.

6. Please include captions for your Supporting Information files at the end of your manuscript, and update any in-text citations to match accordingly. Please see our Supporting Information guidelines for more information: http://journals.plos.org/plosone/s/supporting-information.

RESPONSE: Thank you for your insightful recommendations. A supporting information section has been added at the end of the manuscript, with captions for each supporting information file.

7. Please remove all personal information, ensure that the data shared are in accordance with participant consent, and re-upload a fully anonymized data set.

Note: spreadsheet columns with personal information must be removed and not hidden as all hidden columns will appear in the published file.

Additional guidance on preparing raw data for publication can be found in our Data Policy (https://journals.plos.org/plosone/s/data-availability#loc-human-research-participant-data-and-other-sensitive-data) and in the following article: http://www.bmj.com/content/340/bmj.c181.long.

RESPONSE: Thank you for your insightful recommendations. We checked the dataset to confirm that all personal and indirect identifiers have been removed (not hidden).

8. If the reviewer comments include a recommendation to cite specific previously published works, please review and evaluate these publications to determine whether they are relevant and should be cited. There is no requirement to cite these works unless the editor has indicated otherwise.

RESPONSE: Thank you for your insightful recommendations. We looked at all of the recommended publications. Citations were included only if they were directly relevant to our work, in compliance with PLOS ONE's editorial guidelines.

Point-by-point reply to the reviewers' comments

1. Language and grammar: The language used in the manuscript is clear, but several grammatical and structural issues diminish clarity (for example, "Nurse Professionals who were bothered by a lack of material" – awkward phrasing). Consider professional editing to improve readability. anguage and grammar:

RESPONSE: We are thankful to the reviewer for this valuable suggestion. We carefully reviewed the work for grammar and overall readability. We also carried out a thorough language edit to guarantee clarity and consistency.

2. Measurement of autonomy:

The way 'autonomy' is measured with 5 Likert-type items is suitable, but dividing it into two categories (good vs. poor based on the mean) might oversimplify complex perceptions. The

authors should consider discussing the limitations of using a binary outcome more thoroughly in the discussion section.

RESPONSE: Thank you for your insightful comments and recommendations. We agree on this crucial aspect. In the revised discussion section, we have added a paragraph discussing the potential oversimplification caused by dichotomizing autonomy ratings.

3. Limitations:

Although the limitations are discussed, I recommend emphasizing the cross-sectional design's inability to infer causality and adding a brief comment on potential self-report bias in measuring autonomy.

RESPONSE: Thank you for your comments regarding the limitation section. We extended the Limitations section to emphasize that the cross-sectional design limits causal inference. We also included a note about the risk of self-report bias influencing autonomy evaluation, given that responses were based on participants' subjective views and recalls.

4. Ethics and Data Availability:

The ethics approval and consent are clearly outlined. Data availability also adheres to PLOS ONE policy.

RESPONSE: We appreciate your positive feedback.

5. Contextualization:

The introduction and discussion could benefit from better integration of relevant global literature, especially outside of sub-Saharan Africa, to expand the discussion of autonomy across healthcare systems.

RESPONSE: Thank you for pointing this out. To give a more comprehensive view of nurse autonomy in various healthcare systems, we have examined and incorporated additional material from a variety of international contexts, including research conducted in the United States.
