# Supplementary material for: Work autonomy and its associated factors among nurses working in South Gondar zone public hospitals, Amhara regional state, Northcentral Ethiopia: Institution-based cross-sectional study
Source: PLoS One. 2026 Feb 13;21(2):e0339208. doi: 10.1371/journal.pone.0339208 (PMC12904413; doi:10.1371/journal.pone.0339208)
Supplement: Attachment — Submitted filename: Point plose.docx [file pone.0339208.s003.docx]

**Response to Reviewers**

We appreciate the time and effort that the editors and the reviewers dedicated to providingfeedback on our manuscript and are grateful for the insightful comments and valuableimprovements to our paper. We have incorporated all suggestions made by the reviewers. Please see below for a point-by-pointresponse to the reviewers’ comments and concerns. We are thankful to the reviewer for this valuable suggestion.

**Point-by-point reply to the reviewers’ comments**

**Reviewer #1**

**Abstract**

1.The papers would be benefited if qualitative study is included as it would allow for deeper understanding of the barriers to nurses' autonomy, making a mixed methods approach the most effective way to investigate this issue.

**RESPONSE:** Thank you for your comments and recommendations. We agreed that incorporating qualitative methods allows for deeper understanding of the barriers to nurses' autonomy. While the current study was quantitative, we acknowledge this limitation in the conclusion and limitation section and recommend future studies to consider a mixed-method approach.

2. What is the relevance of stating this unless otherwise the authors believe it has something related with nurses autonomy “It is bordered to the east by the South and North Wollo zones, to the west by Lake Tana and the Bahirdar Liyu zone, to the north by Central Gondar, to the northeast by the Waghimra zone, and to the south by the East and West Gojjam zones. According to data from the South Gondar Zone Administrative Health Bureau, the population of South Gondar is 2,609,823, with 50.1% women and 49.9% men. The majority of the population lives in rural areas, with around 80-85% residing in rural districts and engaging in agriculture. Urbanization is growing, particularly around key towns like Debre Tabor, which is the administrative center of South Gondar, and other urban centers such as Woreta”. The methods need to be sharper and targeted to the goal of the study.

**RESPONSE:** Thank you for your comments and recommendations on improving our manuscript. We omitted the thorough geographic and demographic description of the South Gondar Zone from the revised manuscript since it was not relevant to the study's purpose.

3. While the repeated use of 'data was' may not be a major issue, such minor grammatical errors can distract readers. It would be much better if authors review the paper for grammar throughout.

**RESPONSE:** We are thankful to the reviewer for this valuable suggestion. We appreciate grammatical concerns and clarity; we have revised the entire manuscript for grammatical accuracy.

4. The authors included all nurses in the study area, noting that the study population was smaller than the intended sample size. However, they did not specify the estimated sample size before making this claim. There appears to be a significant inconsistency regarding the study population, sample size, and study design. If the authors were unable to reach their estimated sample size, it is unclear why they did not include nurses working outside hospitals, such as those in health centres within the zone. The use of a census in their methodology need to be clear such as is the census about all nurses in the zone or only those working in hospitals? It appears that a non-random sampling approach was used, focusing only on nurses working in hospitals, while many nurse professionals in the zone work in health centres and were not included. If authors are about to include nurses working in hospital, they need to answer why nurses working in health centres are excluded in the method section.

**RESPONSE:** We are thankful to the reviewer for this valuable suggestion. We have specified the estimated sample size and all suggestions given in the sample size determination section of the revised manuscript based on your comments.

5. How authors define nurse? It would be clearer who is considered nurse in this study and who is included. Registered diploma nurse, BSc nurse, mental health nurse, nurses with advanced training like Perioperative Anaesthesia Nurse and so on. This is very important particularly their work autonomy might be affected by their level of training and scope that authors may consider adjusting in their regression analysis. Perhaps authors need clearly state this in the method section.

**RESPONSE:** We are thankful to the reviewer for this valuable comment. In this study "nurses" refers to all licensed nurse professionals.

6. Do the authors assess whet her the type of job nurses aimed for affects their work autonomy? What percentage of the study participants are working as nurses by choice versus not by choice? This is a sensitive variable that may be better explored through qualitative research and should be examined further for discussion and recommendations.

**RESPONSE:** Thank you for your insightful comments and recommendations. We did not specifically assess whether the type of job nurses aimed for (working as nurses by choice versus not by choice) influenced their work autonomy. We have now acknowledged this limitation in the limitation section of the revised manuscript.

7. What does the mean score refers? Was it the mean score of each item for all participants or scores from the 5 items are summed or averaged first to get an overall autonomy score for each nurse. Then calculate the mean averaged score of the participant. This needs to be clearer in the method section.

**RESPONSE:** **RESPONSE:** Thank you for your comments. We acknowledge our description of how the autonomy score was calculated was not sufficiently clear. We have now clarified the scoring procedure in the methods section.

**Results**

8. This needs to be entirely presented in the methods section “Using logistic regression analysis with odds ratios (ORs) and 95% confidence intervals (CIs), factors related to work autonomy at public hospitals in the South Gonder Zone, Northcentral Ethiopia, were identified. First, the relationship between each independent variable and the result variable was evaluated using bivariable logistic regression. Twelve variables with a p-value below 0.25 were selected as candidates for multivariable logistic regression analysis. Variables with a p-value less than 0.05 in the multivariable logistic regression analysis were considered statistically significant.”

**RESPONSE:** Thank you for your comments. We are now incorporate this in the methods section and remove in the result section to avoid reputations.

**Discussion**

9. There is contradicting results discussed for example authors said “Nurses who reported being bothered by a lack of material during work had 1.8 times higher odds of having good work autonomy (AOR 1.8, 95% CI 1.1–3.0)” while down in the discussion they stated that “findings from this study showed that being bothered by a lack of materials during work is associated with having a lower level of autonomy than their counterparts”. Authors needed to be clearer and careful of interpreting the findings.

**RESPONSE:** Thank you for insightful comments. We have corrected this inconsistency to ensure accurate interpretation of the findings

10. The result showed 53.5 % of nurses had good work autonomy (above the mean score). However, the authors should also consider the reverse perspective, as 46.5% of nurses report poor work autonomy, a substantial proportion that warrants further investigation and needs to be discussed.

**RESPONSE:** Thank you for pointing this out. We have revised the discussion section to highlight 46.5% of nurses report poor work autonomy.
